# Persistent swallowing disorders after extubation in mechanically ventilated patients in ICU: a two-center prospective study

**DOI:** 10.1186/s13613-020-00752-x

**Published:** 2020-10-14

**Authors:** G. Beduneau, V. Souday, J. C. Richard, J. F. Hamel, D. Carpentier, J. M. Chretien, P. Bouchetemble, L. Laccoureye, A. Astier, V. Tanguy, A. Mercat, F. Beloncle, F. Tamion

**Affiliations:** 1 Medical Intensive Care Unit, Normandie Univ, UNIROUEN, EA 3830, Rouen University Hospital, 76000 Rouen, France; 2 Medical Intensive Care Unit, University Hospital of Angers, UNIV Angers, Angers, France; 3 INSERM UMR 955 Eq13, Créteil, France; 4 Department of Biostatistics and Methodology, University Hospital of Angers, UNIV ANGERS, 49000 Angers, France; 5 Medical Intensive Care Unit, Rouen University Hospital, 76000 Rouen, France; 6 Clinical Research Department, University Hospital of Angers, 49000 Angers, France; 7 Otolaryngology-Head and Neck Surgery Department, Rouen University Hospital, 76000 Rouen, France; 8 Otolaryngology-Head and Neck Surgery Department, University Hospital of Angers, UNIV Angers, Angers, France; 9 Medical Intensive Care Unit, Normandie Univ, UNIROUEN, Inserm U1096, Rouen University Hospital, 76000 Rouen, France

**Keywords:** Mechanical ventilation, Prolonged intubation, Swallowing disorder, Mechanical ventilation weaning

## Abstract

**Background:**

Persistent swallowing disorders (SD) are non-pulmonary complications of mechanical ventilation (MV). However, there are few clinical studies on persistent SD in critically ill patients undergoing tracheal intubation for MV. The aim of the present study was to assess the incidence and characteristics of clinical manifestations associated with persistent SD.

**Methods:**

We prospectively evaluated in patients requiring more than 7 days of invasive MV the incidence and characteristics of clinical manifestations related to persistent SD. For this purpose, quality of swallowing was assessed within 24 h after extubation by an experienced physical therapist not directly involved in patient management. Swallowing assessment consisted in a specific standardized test combining a swallowing test and a full clinical evaluation of the cranial nerves involved in swallowing. In patients with SD on the first test, a second test was done within 48 h in order to discriminate between transient and persistent SD.

**Results:**

Among the 482 patients mechanically ventilated more than 7 days, 138 were enrolled in this study. The first test performed 24 h after extubation revealed SD in 35 patients (25%). According to the second test performed 48 h later, SD were considered transient in 21 (15%) and persistent in 14 (10%) cases. Patients with persistent SD were older (66 ± 16 vs 58 ± 15 years), had lower bodyweight at admission (76 ± 15 vs 87 ± 23 kg) and received less often neuromuscular blocking agents (36% vs 66%) compared to patients without or with only transient SD. Patients with persistent SD had longer duration of Intensive Care Unit (ICU) stay after first extubation and longer delay to oral feeding than patients without or with only transient SD, respectively, 11 ± 9 vs 7 ± 6 days and 23 ± 33 vs 5 ± 7 days.

**Conclusions:**

Based on a specific standardized clinical test, 25% of patients mechanically ventilated more than 7 days exhibited clinical manifestations of SD. However, SD were considered as persistent after extubation in only 10% of them. Persistent SD were associated with longer duration of ICU stay after extubation and longer time of enteral feeding.

Trial registration: The study is registered with Clinical Trials (NCT01360580).

## Background

During the last decade, there has been an increasing interest in non-pulmonary complications of mechanical ventilation (MV) in critically ill patients [1]. In particular, psychiatric or cognitive disorders also known as post-traumatic disorders have been addressed in several studies. Physical sequelae directly or indirectly related to MV are a major focus since they represent an unacceptable price to pay while the overall prognosis of ventilation continues to improve.

However, among the complications associated with several days of tracheal intubation, the persistent dysfunction of swallowing has been poorly studied in the literature. Swallowing disorders (SD) could be associated with extubation failure, pneumonia, weight loss, and prolonged intensive care unit (ICU) or in-hospital stay [2]. The literature reports a large but highly variable incidence of SD following extubation, that may be attributed to the huge heterogeneity of methodologies used [3]. Our hypothesis was that the incidence of SD might be frequently overestimated leading to a useless delay to resume oral feeding. The aim of this study was to systematically evaluate the incidence of clinical manifestations related to persistent SD in critically ill patients requiring more than 7 days of invasive MV.

## Methods

### Patients

This prospective study was conducted in the medical ICUs of two university hospitals in France. During a 1-year period (from June 2010 to June 2011), all patients intubated and mechanically ventilated for more than seven days were prospectively screened. Patients with pre-existing SD or a diagnosis known to cause SD (such as stroke, or oropharyngeal surgery), patients unable to participate, or who declined participation for legal reasons (age below 18 years, deprivation of liberty, pregnancy) were excluded. Extubation criteria were assessed daily in every ventilated patient according to the usual protocols of the two departments [4]. A spontaneous breathing trial was performed once these criteria were met. The decision of extubation was left to the discretion of attending physician. Patients were included on the day of extubation, after receiving oral explanation of the study and an information form. The study protocol was approved by the ethics committee of the French Intensive Care Society (Société de Réanimation de Langue Française, SRLF) and by the local Institutional Review Board (CPP Nord-Ouest 1). Results have been partially shown in the congress as an abstract [5].

### Study procedure

Quality of swallowing was assessed within 24 h after extubation. Swallowing assessment consisted in a specific standardized test, combining a full clinical evaluation of the cranial nerves involved in the different stages of swallowing, a swallowing test (Additional file 1), and the Medical Research Council (MRC) scale for muscle strength (Additional file 2). This swallowing assessment was performed by an experienced physical therapist (not directly involved in the care process of the patient) with sequential water test, using a standardized procedure routinely used in the two participating hospitals: first, four teaspoons of gelatinized water or cold, colored compote were ingested, and then four teaspoons of liquid (similarly cold and colored) were also ingested.

The diagnosis of SD was considered when cough occurred immediately after the water swallowing test. In order to detect silent aspiration, the physical therapist observed the patient for 1 min maximum for symptoms such as oxygen desaturation ≥ 5% since the beginning of the test, "wet voice", iterative swallowing or bronchospasm after water swallowing test. Such symptoms also gave rise to a diagnosis of SD.

In patients with SD on the first test, a second test was done 48 h later in order to discriminate between transient and persistent SD. According to the usual protocols of the two ICUs, oral feeding was suspended in patients exhibiting persistent SD. However, decision to resume feeding was left to the discretion of the attending physician. Follow-up was stopped at day 28 and data on reintubation, pneumonia treated by antibiotics and delay to oral feeding were recorded.

### Data collection

The following patients’ characteristics were recorded: age, sex, admission weight, severity of illness evaluated by the Simplified Acute Physiology Score II (SAPSII) at 24 h and Sequential Organ Failure Assessment (SOFA) score at day 1, the Medical Research Council (MRC) scale for muscle strength score at extubation and every week according to the patient’s level of awareness. We also recorded data on intubation: number of exposures, oro or nasotracheal route, ratio between tube diameter and patient size; use of neuromuscular blocking agents during MV; and extubation: planned or not, use of corticosteroids, prokinetics or antacids in the 48 h before extubation. We also recorded data on outcomes, such as need for reintubation, non-invasive ventilation after extubation or tracheotomy. We also recorded antibiotic prescription for pneumonia (until return home within a limit of 28 days) and delay to return to oral feeding (both partial and exclusive).

### Statistics

Data were analyzed using Stata software, version 12.1. Qualitative variables are expressed as percentages and compared using Fisher’s exact test and quantitative variables as means (standard deviation) and compared using Mann–Whitney test. A *p*-value < 0.05 was considered as being statistically significant.

## Results

### Characteristics of population

**Fig. 1:**
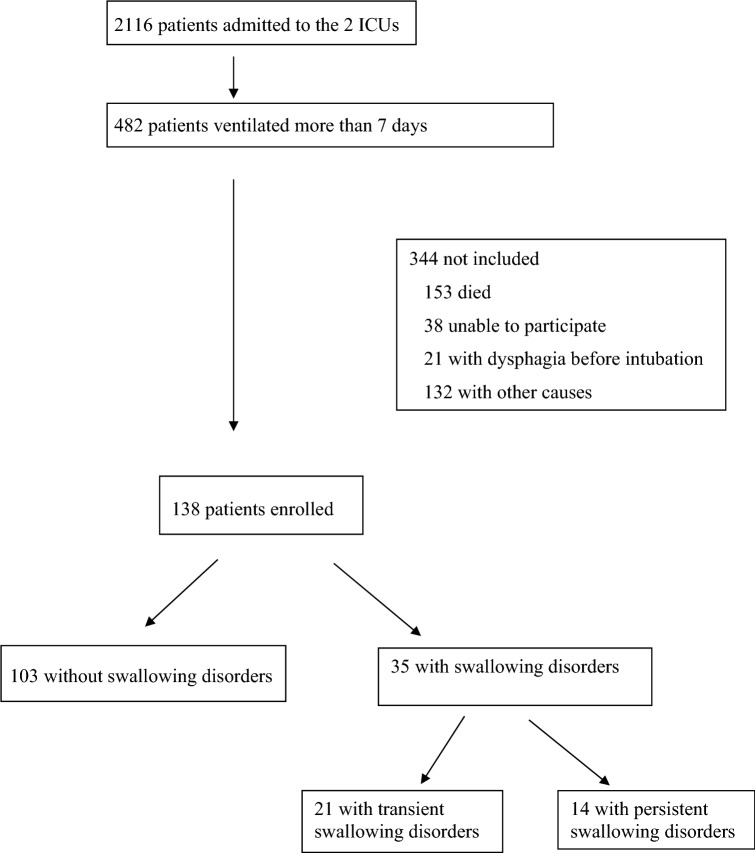
Study flowchart

During the study period, 2116 patients were admitted to the two ICUs. Among 482 consecutive patients with more than seven days of MV, 138 were enrolled in the study (Fig. 1).

Mean age was 58.8 (± 14.9) years. Mean SAPS II was 56 (min = 17, max = 117). Mean duration of MV before swallowing assessment was 15 (± 7.8) days. All patients except three were intubated by oral route.

### Incidence and persistence of swallowing disorders

**Fig. 2:**
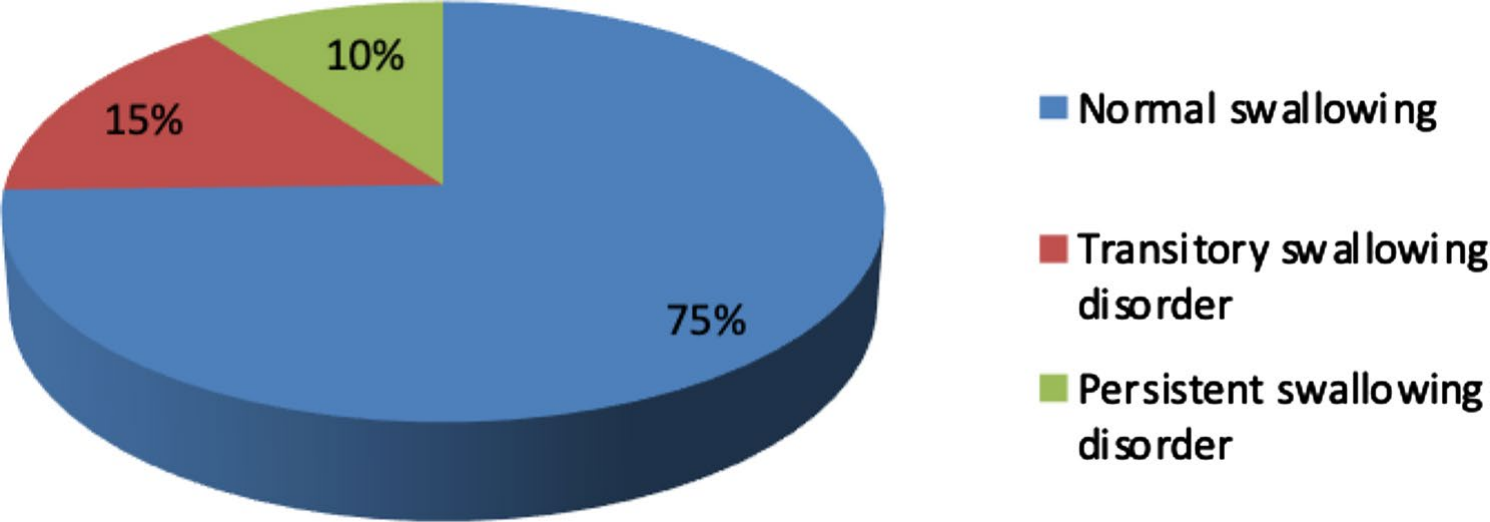
Results of swallowing test

The first test, performed within 24 h after extubation, revealed SD in 35 patients (25%). According to the second test performed within 48 h after the first one (36 to 48 h after), SD was transient in 21 patients (15%) and persistent in 14 patients (10%) (Fig. 2).

### Factors associated with persistent swallowing disorders

Patients exhibiting persistent SD (*N* = 14) were compared with patients with no SD (*N* = 103) and only transient SD (*N* = 21).

Patients with persistent SD were older, had lower body weight at admission and received less often neuromuscular blocking agents during the first days of MV. Injury of XII cranial nerve at extubation was also significantly more frequently observed in patients with persistent SD. These results are presented in Table 1.

**Table 1:** Factors associated with persistent swallowing disorders vs no or transient swallowing disorders

	No or transient SD *n* = 124 (90%)	Persistent SD *n* = 14 (10%)	*p*
Male (*n*, (%))	73 (59)	8 (57)	
Age (years; mean ± SD)	58 ± 15	66 ± 16	*0.039*
SAPS II at admission (mean ± SD)	56 ± 21	58 ± 15	0.395
SOFA score at D1 (mean ± SD)	9 ± 4	8 ± 4	0.283
Admission weight (kg; mean ± SD)	87 ± 23	76 ± 15	*0.033*
Ratio patient size/tube diameter (mean ± SD)	4.5 ± 0.3	4.5 ± 0.2	0.701
Neuromuscular blocking agent (*N*; (%))	81 (66)	5 (36)	*0.039*
Duration of neuromuscular blocking agent (*D*; (mean ± SD)	3 ± 3	1 ± 2	*0.023*
Steroids (*N*; (%))	34 (28)	3 (21)	0.144
Prokinetics during 48 h before extubation (*N*; (%))	7 (6)	1 (7)	0.588
Antacids during 48 h before extubation (*N*; (%))	44 (36)	6 (43)	0.770
Duration of ICU stay (*D*; mean ± SD)	21 ± 11	28 ± 15	*0.073*
Duration of intubation (*D*; mean ± SD)	14 ± 7	17 ± 10	0.253
Duration of NIV (*D*; mean ± SD)	1 ± 4	1 ± 2	0.528
MRC Scale for Muscle Strength score at extubation (mean ± SD)	43 ± 11	37 ± 10	0.057
XII cranial nerve injury at extubation (*N*; (%))	58 (47%)	11 (79%)	*0.045*

*SD* swallowing disorders, *SAPS II* Simplified Acute Physiology Score II, *SOFA score* Sepsis-related Organ Failure Assessment score, *ICU* Intensive Care Unit, *NIV* non-invasive ventilation, *MRC score* Medical Research Council

### Patient outcomes

Outcomes of patients with persistent SD (*N* = 14) were compared to those of patients without persistent SD or only transient SD (*N* = 124). Patients with persistent SD had a longer duration of ICU stay after first extubation and a longer delay to oral feeding. These results are presented in Table 2.

**Table 2:** Patients’ outcomes

	No or transitory SD *n* = 124 (90%)	Persistent SD *n* = 14 (10%)	*p*
Reintubation (*N*, (%))	10 (8)	3 (21)	0.129
Antibiotics for pneumonia after extubation (*N*; (%))	7 (6)	1 (7)	0.597
Duration of exclusive tube feeding (*D*; mean ± SD))	2 ± 5	4 ± 6	0.076
Delay to exclusive oral feeding (*D*; mean ± SD)	5 ± 7	23 ± 33	*0.015*
Duration of ICU stay after first extubation (*D*; mean ± SD)	7 ± 6	11 ± 9	*0.041*
Return to exclusive oral feeding (*N*;(%))	102 (82)	8 (57)	*0.038*

*SD* swallowing disorders, *ICU* Intensive Care Unit

## Discussion

Based on a specific standardized test combining a swallowing test and a clinical evaluation of the cranial nerves involved in swallowing, we found that 25% of patients mechanically ventilated for 7 days or more exhibited clinical evidence of SD. Surprisingly, SD were considered persistent in only 10% of them based on the second test performed 48 h after the first one. Presence of persistent SD was associated with longer duration of ICU stay after extubation and longer time of enteral feeding. SD were not associated with an increased risk of pneumonia and reintubation in the present study.

### Incidence of swallowing disorders

During the last decade, there has been an increasing interest in the literature regarding the long-term outcomes of ICU stay in terms of physical or psychological effects [1]. Among these effects, swallowing dysfunction is considered as a complication of MV that may favor aspiration of oral secretion, with an associated risk of pneumonia, a higher rate of reintubation, malnutrition or dehydration [3, 6–8]. Nevertheless, the incidence of SD among mechanically ventilated patients is difficult to assess and the methodology used in numerous studies is heterogeneous, sometimes non-specific, frequently enrolling specific subgroups of patients. Thereby in a systematic review of 14 highly heterogeneous studies (regarding population enrollment diagnosis strategy), SD incidence ranged from 3 to 62% [3].

### Diagnostic strategy for SD detection

Reliable detection of SD is necessary to decide the optimal time to resume safe oral administration of foods, liquids or oral medications. In addition, erroneous diagnosis of SD may lead caregivers to wrongly postpone oral feeding since the fear of regurgitation is widespread. The screening procedure to systematically detect SD in routine practice must be reliable and easy to perform. Our results from the ICUs of two university hospitals suggest that a pragmatic clinical bedside assessment performed by an experienced caregiver following a systematic process could be implemented in routine practice. Macht et al. published in 2011 a retrospective observational study, using a bedside swallow evaluation [9]. In their large cohort of critically ill patients, they showed that swallowing disorders diagnosed by this clinical method were associated with a composite outcome of pneumonia, reintubation and death. Furthermore, in this study MV for more than seven days was significantly associated with moderate or severe dysphagia. Padovani also described a clinical method of SD diagnosis for critically ill patients, usable by health professionals as a first-line, with steps very similar to ours [10]. More recently, Schefold et al. in a prospective observational trial observed that systematic dysphagia screening performed by trained ICU nurses was positive for 12.4% of patients after extubation [11]. The same authors, in a review published in 2019, highlighted the need for studies assessing a clinical approach to this problem [12].

### Complications and factors associated with swallowing disorders

In our study, patients with persistent SD were older, thinner at admission and received less often neuromuscular blocking agents (36% vs 66%) during the first days of MV compared to patients without or with only transient SD. In their study [13], Macht et al. found in univariate analysis an association between duration of MV and post-extubation dysphagia, but not with age or weight. Although several risk factors have been identified, the underlying mechanisms contributing to dysphagia in ICU patients remain incompletely understood. Most studies report conflicting results that could be explained by selection bias and the small number of patients enrolled [14]. The effect of neuromuscular blocking agents has been poorly analyzed in previous studies. However, we believe that these agents help to prevent laryngeal injury, which is frequently found in patients with prolonged intubation [15] and which may contribute to the onset of SD.

In our study, patients with persistent SD had significantly more frequently injury of XII cranial nerve at extubation. This original finding may be explained as a local injury due to the use of endotracheal tube, or as a complication due to an ICU acquired weakness [16, 17]. In parallel, we observed lower scores of MRC Scale for Muscle Strength at extubation for patients with persistent SD, without reaching significance. It should be noted that the MRC was measured on extubation while the persistent SD was diagnosed on average 72 h later. This could explain that the difference is not significant. Our objective was to determine an association between critical illness polyneuromyopathy and SD, as suggested in recent publications [16–18].

### Patient outcomes

In a non-adjusted analysis, we highlighted, significant differences in duration of ICU stay after extubation and delay to oral feeding between patients with persistent SD and patients without SD or only transient SD. Macht et al. found that the presence of severe post-extubation dysphagia was significantly associated with poor patient outcomes, including pneumonia, reintubation, in-hospital mortality, hospital length of stay, discharge status and surgical placement of feeding tubes. In multivariate analysis, authors found that the presence of moderate or severe dysphagia was independently associated with the composite outcome of pneumonia, reintubation and death [9].

We arbitrarily selected a period of 7 days of invasive MV to keep only prolonged ventilation. Interestingly, Macht et al. [9] showed after multivariate analysis, that mechanical ventilation for more than 7 days was significantly associated with moderate or severe dysphagia. These results corroborate a posteriori our criteria for duration of MV.

The design of our study allowed us to discriminate between transient and persistent SD. In our series, the persistence of SD in only 14 among 35 patients with post-extubation SD indirectly suggests that oral feeding could have been resumed earlier in almost 60% of patients who were wrongly identified as at risk for aspiration pneumonia. This hypothesis warrants further more powerful studies to assess the clinical impact of such a strategy.

## Limitations of the study

The methodology of the present study is based on a clinical evaluation and the lack of concomitant anatomic examination (dynamic laryngoscopy) may have resulted in an overestimation of the number of real anatomic injuries. Even if the main purpose was to assess clinical evidence of SD, it would have been interesting to evaluate its association with anatomic lesions. However, the use of a screening stage based on an easy-to-implement clinical assessments is recommended in the recent review of Perren et al. [12]. This study could be considered as underpowered to identify associated risk factors of persistent SD since, among the 138 patients enrolled in our study, only 35 exhibited SD. Nevertheless, to our knowledge, this is the largest series of patients mechanically ventilated for more than 7 days with systematic assessment of swallowing at extubation. At the same time, we did not find any significant difference in rate of reintubation or antibiotic prescription for pneumonia after extubation. One should keep in mind that the team in charge of the patient was not blinded to the results of swallowing assessment, which may have contributed to the low number of complications related to SD in our study. As expected, the duration of tube feeding was significantly longer in patients with persistent SD. Finally, we were unfortunately not able to collect the data concerning the conditions and the complications of the intubation procedure.

## Conclusions

In this cohort, clinical evidence of SD was observed in 25% of patients mechanically ventilated for more than 7 days. Interestingly only 40% of them exhibited persistent SD within a maximum of 3 days after extubation. Associated factors were higher age, lower body weight, and lower use of neuromuscular blocking agents during the period of mechanical ventilation. In this non-adjusted analysis, SD were associated with longer duration of stay in ICU after extubation and longer time of enteral feeding.

A systematic clinical evaluation of swallowing after invasive ventilation is feasible and should be repeated after 48 h to limit the effect of a useless delay before resuming oral feeding.

## Supplementary information

**Additional file 1.** Clinical evaluation and swallowing test.

**Additional file 2.** Medical Research Council (MRC) Scale for Muscle Strength.

## Data Availability

The datasets analyzed during the current study are available from the corresponding author on reasonable request.

## Abbreviations

MV: Mechanical ventilation
SD: Swallowing disorders
ICU: Intensive Care Unit
MRC: Medical Research Council
SAPSII: Simplified Acute Physiology Score II
SOFA: Sequential Organ Failure Assessment
