# Supplementary material for: Persistent swallowing disorders after extubation in mechanically ventilated patients in ICU: a two-center prospective study
Source: Ann Intensive Care. 2020 Oct 14;10:138. doi: 10.1186/s13613-020-00752-x (PMC7560673; doi:10.1186/s13613-020-00752-x)
Supplement: Supplementary file 1 — Additional file 1. Clinical evaluation and swallowing test. [file 13613_2020_752_MOESM1_ESM.docx]

Additional material 1:

**CLINICAL EVALUATION AND SWALLOWING TEST**

| Mouth opening (V,VII) | 🔿 possible 🔿 limited 🔿impossible 🔿 non evaluated |
| --- | --- |
| Mouth closing (V,VII) | 🔿 possible 🔿 limited 🔿impossible 🔿 non evaluated |
| Facial expression (VII) | 🔿 possible 🔿 limited 🔿impossible 🔿 non evaluated |
| Mastication (V) | 🔿 possible 🔿 limited 🔿impossible 🔿 non evaluated |
| Glottic closure and cough | 🔿 possible 🔿 limited 🔿impossible 🔿 non evaluated |
| Lingual mobility (XII) : ability  To put out her/his tongue | 🔿 possible 🔿 limited 🔿impossible 🔿 non evaluated |
| To move from right to left | 🔿 possible 🔿 limited 🔿impossible 🔿 non evaluated |
| To move from top to bottom | 🔿 possible 🔿 limited 🔿impossible 🔿 non evaluated |
| To click her/his tongue | 🔿 possible 🔿 limited 🔿impossible 🔿 non evaluated |
| Tonicity of the base of the tongue (XII) | 🔿 possible 🔿 limited 🔿impossible 🔿 non evaluated |
| Soft palate closure (IX) | 🔿 possible 🔿 limited 🔿impossible 🔿 non evaluated |
| Gag reflex (IX) | 🔿 possible 🔿 limited 🔿impossible 🔿 non evaluated |
| Laryngeal ascent (XI) | 🔿 possible 🔿 limited 🔿impossible 🔿 non evaluated |

| Voice | 🔿 normal  🔿 nasal  🔿 « wet »  🔿 dysphonia  🔿 aphonia  🔿 other [______________________] | |
| --- | --- | --- |
| Pain in swallowing | | 🔿 yes 🔿 no |
| Labial sensitivity (V) | | 🔿 yes 🔿 no |
| Cheek sensitivity (V) | | 🔿 yes 🔿 no |
| Tactil lingual sensitivity (V) | | 🔿 yes 🔿 no |
| Taste lingual sensitivity (VII, IX) | | 🔿 yes 🔿 no |
|  | |  |
| **Swallowing test** | | |
| Primary subglottic penetration | | 🔿 yes 🔿 no |
| Secondary subglottic penetration | | 🔿 yes 🔿 no |
