# Supplementary material for: Persistent swallowing disorders after extubation in mechanically ventilated patients in ICU: a two-center prospective study
Source: Ann Intensive Care. 2020 Oct 14;10:138. doi: 10.1186/s13613-020-00752-x (PMC7560673; doi:10.1186/s13613-020-00752-x)
Supplement: Supplementary file 2 — Additional file 2. Medical Research Council (MRC) Scale for Muscle Strength. [file 13613_2020_752_MOESM2_ESM.docx]

Additional material 2:

**Medical Research Council (MRC) Scale for Muscle Strength**

The patient’s effort is graded on a scale of 0-5:

- Grade 5: Muscle contracts normally against full resistance.
- Grade 4: Muscle strength is reduced but muscle contraction can still move joint against resistance.
- Grade 3: Muscle strength is further reduced such that the joint can be moved only against gravity with the examiner’s resistance completely removed.
- Grade 2: Muscle can move only if the resistance of gravity is removed.
- Grade 1: Only a trace or flicker of movement is seen or felt in the muscle or fasciculations are observed in the muscle.
- Grade 0: No movement is observed.

| **Right** |  | **Left** |
| --- | --- | --- |
|  | Abduction of the arm |  |
|  | Flexion of the forearm |  |
|  | Extension of the wrist |  |
|  | **Total upper limb** |  |
|  | Flexion of the thigh |  |
|  | Extension of the leg |  |
|  | Dorsal flexion of the foot |  |
|  | **Total lower limb** |  |

Total (from 0 to 60) = sum of the total scores of the 4 limbs
